# IDENTIFYING RISK PERCEPTION ON FALL OF COMMUNITY-DWELLING OLDER ADULTS USING PHOTOS

**DOI:** 10.1093/geroni/igae098.2717

**Published:** 2024-12-31

**Authors:** Ji Yeon Lee, Gwang Suk Kim, Min Kyung Park, Layoung Kim, Jae Jun Lee

**Affiliations:** Inha University, Incheon, Republic of Korea; Yonsei University, Seoul, Republic of Korea; Yonsei University College of Nursing, Seoul, Republic of Korea; Yonsei University, Seoul, Republic of Korea; Yonsei University, Seoul, Republic of Korea

## Abstract

Most falls in older adults occur within the home, especially in a bathroom. To prevent indoor falls, increasing fall risk perception is necessary; however, scarce research explored the risk perception of indoor falls in community-dwelling older adults. This study was an exploratory mixed-methods research using photos. We recruited 10 experts with extensive experience in home environmental assessment/modification and nine older adults with fall experience in home bathrooms within the last two years. All participants evaluated six-bathroom photos through one-to-one interviews with a structured questionnaire, which was based on the health belief model: individual perception, modifying factors, and likelihood of action. The mean age of the expert was 45.8 and 50% was female. The average years in home environment modification/assessment was 10.9 years. Regarding older adults, the mean age was 75.1 and 66.7% were female. The number of diagnosed diseases was 1.86 and many reported bad vision (66.7%) and hearing loss (44.5%). The expert group had a higher risk perception and found extensive risk factors including doorsill, door direction, slippery floor, improper toilet height, narrow space around the toilet, no washbasin, handrails in bathtub and entry, bathtub height, and not organized environment. Older adults showed lower-risk perception in all pictures and risk factors included doorsill, slippery floor, no washbasin, no handrail, and not organized environment. Our findings showed older adults with a fall history have a lower-risk perception regarding environmental hazards. Therefore, education is needed to increase risk perception, and assessment/intervention of the home environment is necessary to prevent further falls.

